# Supplementary material for: The dynamic proteome of influenza A virus infection identifies M segment splicing as a host range determinant
Source: Nat Commun. 2019 Dec 4;10:5518. doi: 10.1038/s41467-019-13520-8 (PMC6892822; doi:10.1038/s41467-019-13520-8)
Supplement: Supplementary file 1 — Supplementary Information [file 41467_2019_13520_MOESM1_ESM.pdf]

Supplementary Figures 1-4

The dynamic proteome of influenza A virus infection identifies M segment splicing as a host range determinant

Bogdanow et al.

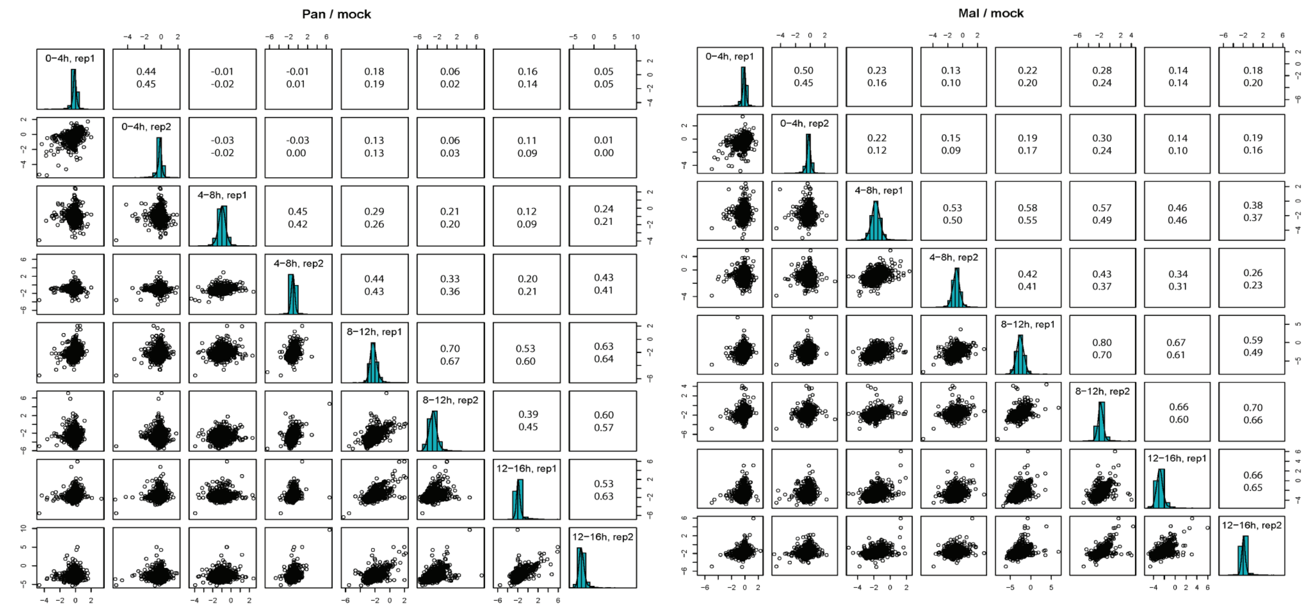

Supplementary Figure 1.
Spearman's (top number) and Pearson's correlation coefficients (bottom number) of biological (label-swap) replicates for SILAC ratios Pan/mock (left) and Mal/mock (right). In the first replicate SILAC H labelled cells were infected with the Mal strain, SILAC M labelled cells infected with the Pan strain, and SILAC L cells were mock infected. In the second replicate, SILAC M labelled cells were infected with the Mal strain, SILAC H labelled cells were infected with the Pan strain and SILAC L cells were mock infected. Differences in the slope may be due to label differences.
For this reason ratios were averaged.

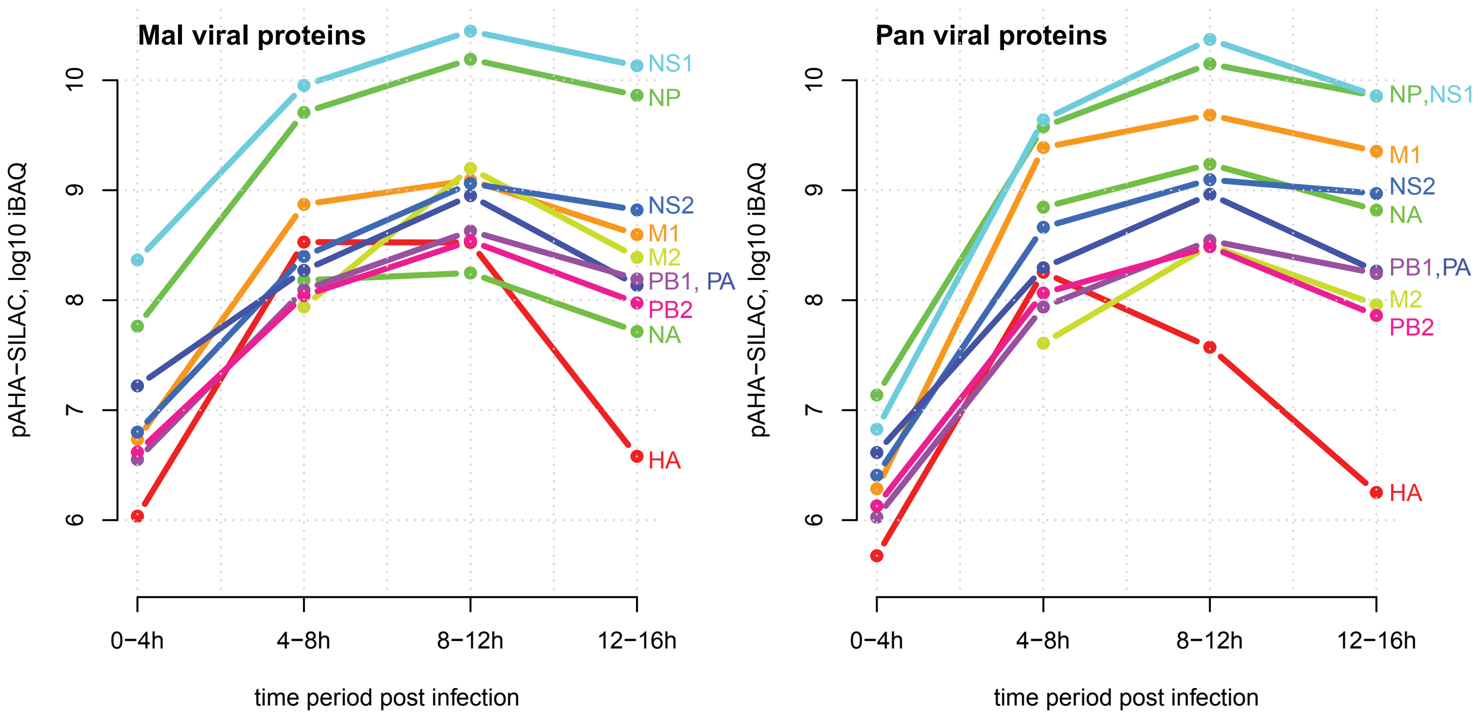

Supplementary Figure 2.
Viral protein expression kinetics. iBAQ-based absolute quantification of protein synthesis across infection for the indicated 10 viral proteins of strain Mal (left) or strain Pan (right). Proteins quantified in only one replicate were removed.

a

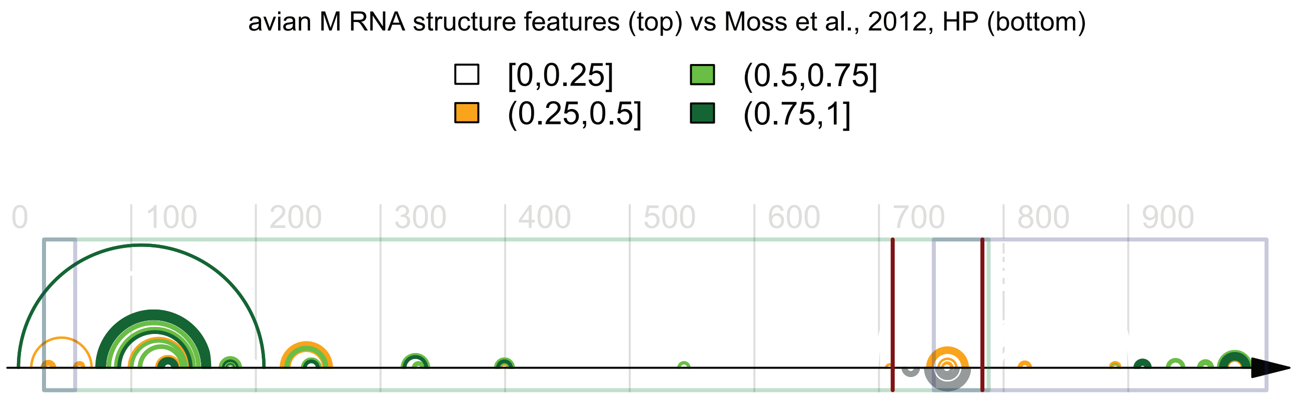

b

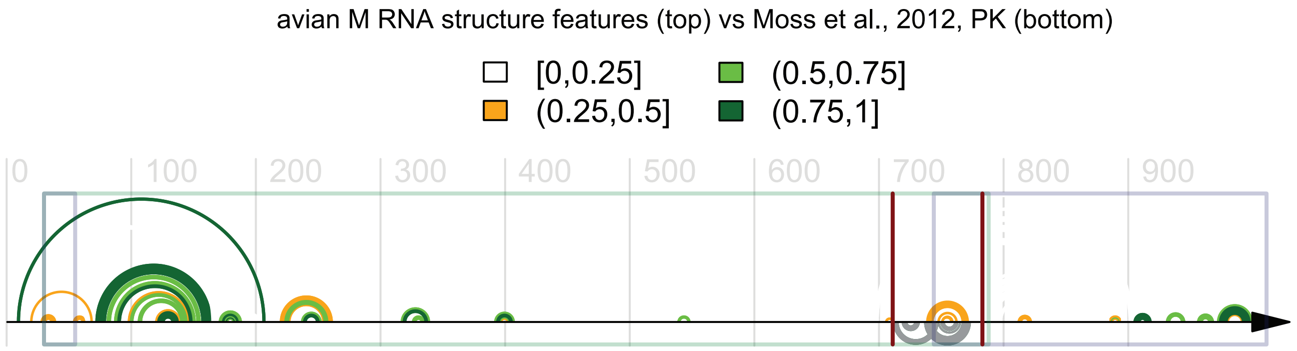

Supplementary Figure 3.
Agreement between the RNA secondary structure predicted by RNA-Decoder for the M mRNA for the avian-adapted influenza strains and the RNA structure features predicted by Moss et al.. Agreement with the hairpin (HP) in a and with the pseudoknot (PK) in b. Colour-coding of base-pairs according to the corresponding, estimated base-pairing probabilities, see also legend of Figure 5. The single exon containing the contiguous open-reading frame (ORF) of the M1 splice variants is indicated by a green box, the two exons corresponding to the M2 splice variant are shown as two blue boxes. Note that there are two regions where the ORFs of the splice variants overlap. The first one corresponds to the first exon of the M2 splice variant where the two ORFs are in sync. The second one corresponds to the region of overlap between the 3' end of the long M1 ORF and the 5' start of the second exon of the M2 splice variant. In that region, the two ORFs are out of sync, implying a particularly strong constraint due to the two, intertwined amino-acid contexts. The two vertical bars indicate the start and end position of the region that was used for chimeric constructs around the 3' splice site.

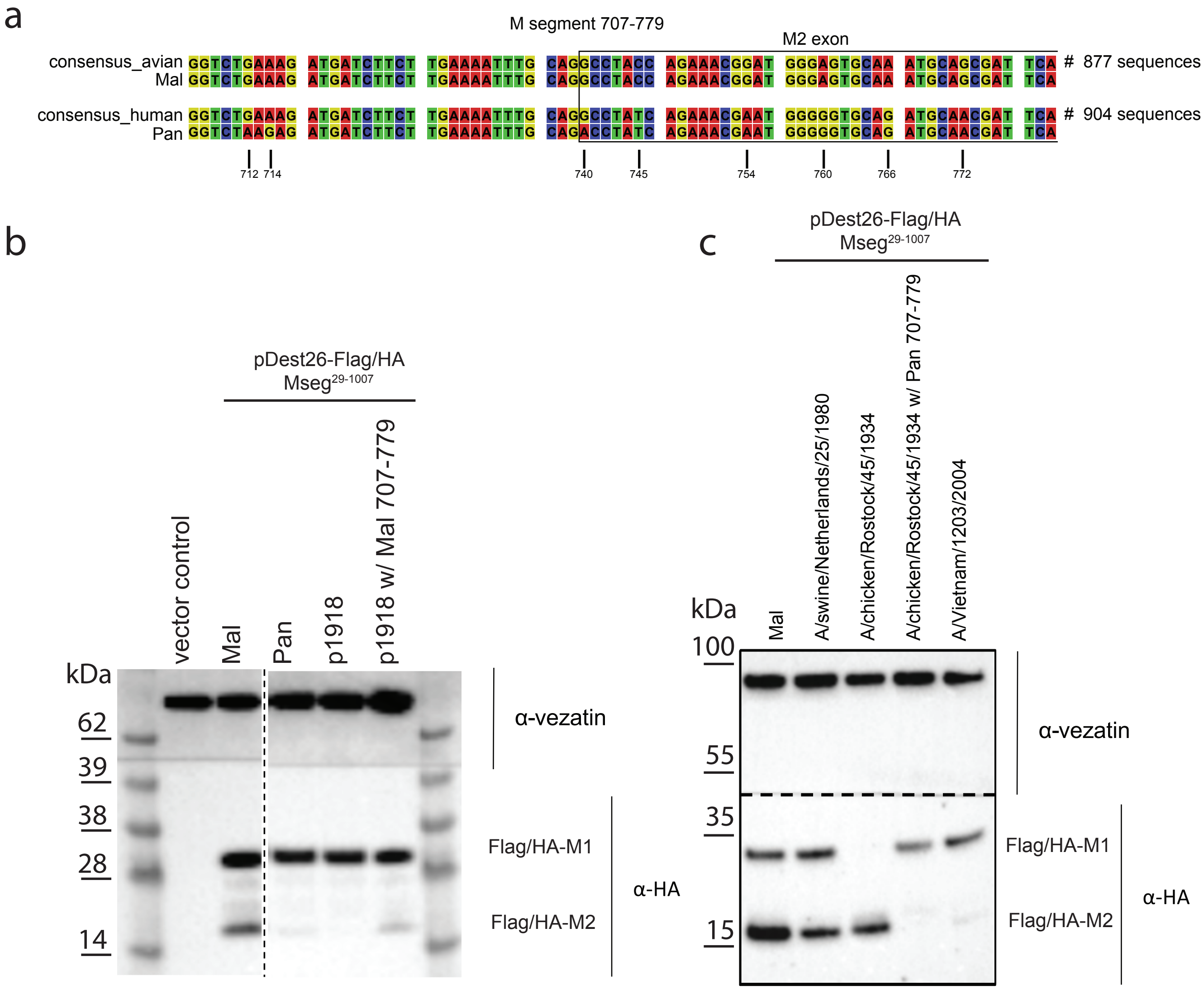

Supplementary Figure 4.

M segment RNA splicing with constructs from other isolates. **a** Multiple sequence alignment of the nucleotide sequences of strain Pan, Mal and the consensus sequences of avian and human IAVs in the M segment 3' splice site region. **b,c** A549 cells were transfected with the indicated expression constructs and M1 / M2 expression was assessed by immunoblotting against HA-antigen. Vezatin staining serves as a loading control.
