# Supplementary material for: The dynamic proteome of influenza A virus infection identifies M segment splicing as a host range determinant
Source: Nat Commun. 2019 Dec 4;10:5518. doi: 10.1038/s41467-019-13520-8 (PMC6892822; doi:10.1038/s41467-019-13520-8)
Supplement: Supplementary file 3 — Reporting Summary [file 41467_2019_13520_MOESM3_ESM.pdf]

nature research

Corresponding author(s): Matthias Selbach

Reporting Summary

Nature Research wishes to improve the reproducibility of the work that we publish. This form provides structure for consistency and transparency in reporting. For further information on Nature Research policies, see Authors & Referees and the Editorial Policy Checklist.

Statistical parameters

When statistical analyses are reported, confirm that the following items are present in the relevant location (e.g. figure legend, table legend, main text, or Methods section).

n/a	Confirmed	
☐	☒	The exact sample size (n) for each experimental group/condition, given as a discrete number and unit of measurement
☐	☒	An indication of whether measurements were taken from distinct samples or whether the same sample was measured repeatedly
☐	☒	The statistical test(s) used AND whether they are one- or two-sided<br>*Only common tests should be described solely by name; describe more complex techniques in the Methods section.*
☒	☐	A description of all covariates tested
☐	☒	A description of any assumptions or corrections, such as tests of normality and adjustment for multiple comparisons
☐	☒	A full description of the statistics including central tendency (e.g. means) or other basic estimates (e.g. regression coefficient) AND variation (e.g. standard deviation) or associated estimates of uncertainty (e.g. confidence intervals)
☒	☐	For null hypothesis testing, the test statistic (e.g. F, t, r) with confidence intervals, effect sizes, degrees of freedom and P value noted<br>*Give P values as exact values whenever suitable.*
☒	☐	For Bayesian analysis, information on the choice of priors and Markov chain Monte Carlo settings
☒	☐	For hierarchical and complex designs, identification of the appropriate level for tests and full reporting of outcomes
☐	☒	Estimates of effect sizes (e.g. Cohen's d, Pearson's r), indicating how they were calculated
☐	☒	Clearly defined error bars<br>*State explicitly what error bars represent (e.g. SD, SE, CI)*

Our web collection on statistics for biologists may be useful.

Software and code

Policy information about availability of computer code

Data collection	FluDB, www.fludb.org

Data analysis

Maxquant version 1.6.0.1 and 1.5.2.8
R version 3.5.1
Rstudio version 1.0.136
Metascape
ImageJ
MUSCLE
Bowtie2 (version 2.1.0)
Tophat2 (v2.0.10)
Cufflinks (v2.2.1)
Rchie
PhyML
RNA decoder
VARNA

For manuscripts utilizing custom algorithms or software that are central to the research but not yet described in published literature, software must be made available to editors/reviewers upon request. We strongly encourage code deposition in a community repository (e.g. GitHub). See the Nature Research guidelines for submitting code & software for further information.

Data

Policy information about availability of data

All manuscripts must include a data availability statement. This statement should provide the following information, where applicable:

- Accession codes, unique identifiers, or web links for publicly available datasets
- A list of figures that have associated raw data
- A description of any restrictions on data availability

Proteomic data relating to the pAHA-SILAC and pSILAC experiments was uploaded to ProteomeXchange Consortium via the PRIDE partner repository with the dataset identifier PXD011321 (pAHA-SILAC) and PXD015475, PXD015474 (pSILAC) RNA-sequencing data is publicly available under PRJNA495615 [https://www.ncbi.nlm.nih.gov/sra/PRJNA495615]. The source data underlying Figs 1c,d, 2a-d,f-h, 3a,d, 4a,b,d,f, 5c-g, 6b-f, S2, S4a-c are available as source data file.

Field-specific reporting

Please select the best fit for your research. If you are not sure, read the appropriate sections before making your selection.

☒ Life sciences ☐ Behavioural & social sciences ☐ Ecological, evolutionary & environmental sciences

For a reference copy of the document with all sections, see nature.com/authors/policies/ReportingSummary-flat.pdf

Life sciences study design

All studies must disclose on these points even when the disclosure is negative.

Sample size	N/A
Data exclusions	no data were excluded
Replication	AHA-SILAC samples were biological duplicates (label-swaps)<br>RNA-seq samples were biological duplicates<br>pSILAC samples were either single samples or label-swap duplicates
Randomization	samples were not randomized
Blinding	investigators were not blinded

Reporting for specific materials, systems and methods

Materials & experimental systems

n/a	Involved in the study
☒	☐ Unique biological materials
☐	☒ Antibodies
☐	☒ Eukaryotic cell lines
☒	☐ Palaeontology
☒	☐ Animals and other organisms
☒	☐ Human research participants

Methods

n/a	Involved in the study
☒	☐ ChIP-seq
☐	☒ Flow cytometry
☒	☐ MRI-based neuroimaging

Antibodies

Antibodies used	HA (clone 3F10, Roche), vezatin (clone B-1, SantaCruz), M1 (clone GA2B, BioRad), M2 (polyclonal, RRID: AB_2549706, Thermo Fisher)
Validation	Recombinant proteins with epitopes for the antibodies were transiently expressed in eukaryotic cells. Antibody validation was based on specifically detecting these recombinant proteins.

Eukaryotic cell lines

Policy information about cell lines

Cell line source(s)	ATCC
Authentication	cell lines were not further authenticated
Mycoplasma contamination	All cell lines were tested negative for Mycoplasma contamination
Commonly misidentified lines (See ICLAC register)	N/A

Flow Cytometry

Plots

Confirm that:

☐ The axis labels state the marker and fluorochrome used (e.g. CD4-FITC).

☐ The axis scales are clearly visible. Include numbers along axes only for bottom left plot of group (a 'group' is an analysis of identical markers).

☐ All plots are contour plots with outliers or pseudocolor plots.

☐ A numerical value for number of cells or percentage (with statistics) is provided.

Methodology

Sample preparation	MDCKII cells were infected with IAV, 5hpi cells were harvested and stained for NP antigen
Instrument	FACSCanto II flow cytometer (BD Biosciences)
Software	FACSDiva (BD Biosciences)
Cell population abundance	N/A
Gating strategy	A dot plot was created displaying on a linear scale the forward light scatter (FSC) and sideward light scatter (SSC) of measured particles. An FSC threshold was set for exclusion of cell debris. A region R1 was set that excludes cell doublets and aggregates from further analysis. The R1 population was analyzed on a separate dot plot for FITC fluorescence of infected cells. Non-infected cells were used to discriminate FITC-positive, NP-expressing cells from background fluorescence. A region R2 was set to gate only FITC-positive cells and calculate their proportion of the parental population R1. This percentage was used to calculate the fluorescence forming units (FFU) in the virus inoculum.

☐ Tick this box to confirm that a figure exemplifying the gating strategy is provided in the Supplementary Information.
