# Supplementary material for: The dynamic proteome of influenza A virus infection identifies M segment splicing as a host range determinant
Source: Nat Commun. 2019 Dec 4;10:5518. doi: 10.1038/s41467-019-13520-8 (PMC6892822; doi:10.1038/s41467-019-13520-8)
Supplement: Supplementary file 4 — Description of Additional Supplementary Files [file 41467_2019_13520_MOESM4_ESM.pdf]

Description of Additional Supplementary Files

File Name: Supplementary Data 1
Description: protein level data virus and host

File Name: Supplementary Data 2
Description: Enriched gene ontology terms across the course of infection

File Name: Supplementary Data 3
Description: Nucleotide and amino acid differences at the 3' splice site region
